# Potential value of differentially expressed circular RNAs derived from circulating exosomes in the pathogenesis of rat spinal cord injury

**DOI:** 10.3389/fnins.2022.1003628

**Published:** 2022-11-11

**Authors:** Chunfang Zan, Jianan Li, Fengsong Lin, Zengliang Wang

**Affiliations:** ^1^Division of Vascular Biology, Institute for Stroke and Dementia Research (ISD), LMU Klinikum, Ludwig-Maximilian-University (LMU), Munich, Germany; ^2^Department of Orthopedics, Tianjin Hospital, Tianjin, China

**Keywords:** spinal cord injury, exosome, ncRNA, circRNA, miRNA, mRNA, ceRNA

## Abstract

Spinal cord injury (SCI) remains one kind of devastating neurological damage, and specific molecular mechanisms involved need to be understood deeply. Currently, circular RNAs (circRNAs), as a newly discovered type of non-coding RNAs (ncRNAs), have been under active investigation. Through functional interactions with disease-associated microRNAs (miRNAs), exosome-derived circRNAs have been extensively implicated in various organ pathogenesis. Nevertheless, the functional involvement of circulating circRNAs in SCI onset, progression as well as repair remains poorly explored until now. Of note, there still lacks clinical and experimental evidence in this regard. To obtain some relevant knowledge in this field, this study was originally designed to have a general overview of differentially expressed circRNAs derived from circulating exosomes in SCI rats in comparison with the control rats. It turned out that 709 types of downregulated circRNAs and 346 kinds of upregulated circRNAs were preliminarily screened out. Functional enrichment analyses including kyoto encyclopedia of genes and genomes (KEGG) pathway and gene ontology (GO) were performed to evaluate the possible biological functions of upregulated as well as downregulated circRNAs involved in SCI. Furthermore, five types of upregulated circulating circRNAs including chr4:208359914–208362182+, chr15:20088296–20092102+, chr1:175098934– 175134845–, chr1:175099657– 175128203–, and chr1:175104454– 175134845–, and plus five kinds of downregulated circulating circRNAs including chr11:74154652– 74159524–, chr12:45412398– 45412635–, chr7:137630261– 137648924–, chr6:6280974–6281188+, and chr4:225251864–225254087+, were verified through reverse transcription-polymerase chain reaction (RT-PCR). At last, taking these differentially expressed circRNAs in the center, the circRNA-miRNA-mRNA gene interaction network was constructed to predict the possible functionalities of circRNAs in SCI through anticipating specific interactive miRNAs, giving new insights into how circRNAs contribute to this pathological process. Taken together, these findings suggest the possible involvement and functional significance of circRNAs in SCI.

## Introduction

Spinal cord injury (SCI) remains a catastrophic injured condition for humans, and causes impaired mobility and even permanent neurological deficits, thereby bringing serious social and economic burdens (Ahuja et al., 2017; David et al., 2019; Zipser et al., 2022). Given that there is still a lack of effective therapies for SCI until now, fundamental research is under active investigation in order to provide some promising targets for controlling SCI progression, exacerbation as well as recovery (Yamazaki et al., 2020; Kopper and Gensel, 2021). In terms of mechanisms, the pathological changes of the injured spinal cord are characterized by traumatic injury, ischemia, chronic neuroinflammation, and redox imbalance (Anjum et al., 2020; Alcántar-Garibay et al., 2022). Recently, non-coding RNAs (ncRNAs), for example, microRNAs (miRNAs) and long non-coding RNAs (lncRNAs), have been identified as pivotal players in SCI (Kimura et al., 2021; Liu et al., 2022). Of note, the knockdown of certain lncRNAs such as TUG1, XIST, and ZFAS1 has shown a beneficial effect on SCI development through mouse as well as rat model studies (Chen Y. et al., 2021; Wu et al., 2021; Zhong et al., 2021), indicating the functional involvement and preclinical significance of these ncRNAs in SCI.

Distinct from traditional linear RNAs, circular RNAs (circRNAs), as a new class of ncRNAs, are more stable, not easy to degrade, and not affected by RNA exonuclease due to their unique closed annular structure (Saaoud et al., 2021; Liu and Chen, 2022). In the functional sense, circRNAs have been found to regulate transcription, splicing, cytoplasmic mRNA stability and translation, interference with signaling pathways, etc. Of interest, circRNAs contain plenty of miRNA binding sites and thereby act as miRNA sponges, further counteracting the inhibitory effects of miRNAs on their target genes and meanwhile upregulating their corresponding target gene expression (Liu and Chen, 2022; Misir et al., 2022). This regulatory process involves the competing endogenous RNA (ceRNA) mechanism. Especially, the translation of circRNAs can produce some novel isoforms of proteins, which further determines their clinical significance in human diseases (Wen et al., 2022).

On the one hand, exosomes, as mediators of cell-to-cell communication as well as vehicles of circRNAs and other ncRNAs, are key regulators in specific molecular signaling (Isaac et al., 2021). On the other hand, circRNAs are one class of the most abundant components in exosomes (Li et al., 2021; Jafari et al., 2022). Therefore, exosomal circRNAs attract much attention from researchers in the context of SCI. In a similar vein, Han et al. (2022) gave a comprehensive review about separated/cooperative biological functions of circRNAs and exosomes, highlighting the potential of these exosomal circRNAs in disease states. So far, exosomal circRNAs have been found to participate in tumorigenesis, and cardiovascular inflammation as well as metabolic disorders through targeting the corresponding miRNA-mediated axis (Li et al., 2021; Lin et al., 2021). Even so, the role of exosomal circRNAs in the injured spinal cord has not been well elucidated, which needs to be explored further.

For this reason, we mainly focus on circulating exosome-derived circRNAs in this study, and aim to investigate their potential role and relevant functions in the context of SCI. To this end, we utilized rat SCI model and collected exosomes, procedures and results of which have been well described in our recently published paper (Li et al., 2023). Based on this, exosomal circRNAs were obtained, functional enrichment analyses including kyoto encyclopedia of genes and genomes (KEGG) pathway and gene ontology (GO) ontology were performed, and several differentially expressed circRNAs were confirmed through reverse transcription-polymerase chain reaction (RT-PCR). Furthermore, the interaction network of circRNA-miRNA-mRNA would better explain the predicted functions of exosomal circRNAs in the injured spinal cord based on the ceRNA mechanism.

## Materials and methods

### Rat spinal cord injury model establishment and exosome extraction and identification

The successful establishment of rat SCI model, the isolation of blood exosomes, and the identification of exosomes were detailedly described in our recently published paper (Li et al., 2023). In brief, six adult female rats were randomly divided into two groups, the experimental group (EG) in which SCI surgery was performed on rats, and the control group (CG) in which the skin was injured on rats. Both cohorts of rats were maintained under the same feeding conditions before and after surgery. After 24 h, blood was collected and exosomes were extracted by using the density gradient centrifugation. Then, the classical characteristics of isolated exosomes were verified through Western blot, electron microscopy, and nanoparticle tracking analysis (NTA). Related data on this part would be referred to our previous publication (Li et al., 2023).

### Exosomal RNA isolation

According to the manufacturer’s protocol, Trizol reagent (Thermo Fisher Scientific, Waltham, MA, USA) was used to obtain high-quality RNA from circulating exosomes. Concentrations of different RNA samples were measured through using Nanodrop Spectrophotometer (Thermo Fisher Scientific, Waltham, MA, USA). Then the quantitative control as well as the integrity control were performed for isolated RNA prior to experiments in the next step.

In the process of quantitative control, the purity of RNA was evaluated by using OD260/OD280 values. The final results would be considered as “Pass”, if these OD260/OD280 values are within the range between 1.8 and 2.1, suggesting isolated RNA has high quality and can be used further.

Regarding the integrity control, 1% agarose gel (Thermo Fisher Scientific, Waltham, MA, USA) was utilized to assess the integrity of isolated RNA in this study.

### RNA library construction and RNA sequencing

Prior to RNA library construction, ribosomal RNA (rRNA) needs to be completely removed from total RNA. To this end, NEBNext rRNA Depletion Kit (New England Biolabs, Ipswich, MA, USA) was used according to the producer’s protocol. Following this, the RNA library was then constructed through using NEBNext^®^ Ultra™ II Directional RNA Library Prep Kit (New England Biolabs, Ipswich, MA, USA) according to the standard procedures. Furthermore, RNA library was quality-controlled and then quantified *via* a BioAnalyzer 2100 system (Agilent Technologies, Santa Clara, CA, USA). In the end, circRNA sequencing was conducted on an illumina HiSeq sequencer from Cloud-Seq Biotech (Shanghai, China). All raw data were uploaded to GEO repository as the GSE213561 study (GSM6589180; GSM6589181; GSM6589182; GSM6589183; GSM6589184; GSM6589185), and the link is as follows: https://www.ncbi.nlm.nih.gov/geo/query/acc.cgi?acc=GSE213561.

### RNA sequencing data analysis

As described in the above part, raw data which contain original paired-end reads were initially acquired from Illumina HiSeq 4000 systems (Cloud-Seq Biotech, Shanghai, China). Through using Cutadapt software,^1^ 3′ adaptor was modified, and low-quality reads were removed in order to keep high-quality reads for further analysis (Han et al., 2020). To display relative levels of these circulating circRNAs, these modified reads were analyzed. HISAT2 software^2^ was used to make an alignment of these pre-selected reads to the human reference genome. Next, according to gene transfer format (GTF) files which were obtained from the Ensembl database,^3^ the expression levels of circRNAs, characterized by the fragments per kilobase of exon model per million mapped fragments (FPKM), were calculated *via* Cuffdiff software (Trapnell et al., 2010). Following this, specific fold changes and corresponding *P*-values were calculated based on the FPKM index, and differentially expressed circRNAs from circulating exosomes were eventually determined.

Moreover, in order to predict their probably involved pathways and functional processes, KEGG pathway analysis^4^ and GO analysis^5^ were performed for circulating upregulated as well as downregulated circRNAs, respectively (Kanehisa and Sato, 2020). The value –log10(*P*-value) was calculated and presented as the enrichment score. For both KEGG pathway and GO analysis, fold change > 2 and *P*-value < 0.05 were taken as the threshold of the differential expression of these circRNAs.

### Determination of differentially expressed circular RNAs through reverse transcription-polymerase chain reaction

Next, we would like to separately verify the top ten dysregulated circulating circRNAs in SCI rats, which were revealed by RNA sequencing previously. To this end, RT-PCR was utilized here to show the relative levels of these 10 circulating exosomal circRNAs from both group rats. As mentioned in the above part, Trizol reagent was used to isolate RNA, and then cDNA was synthesized by utilizing SuperScript™ IV First-Strand synthesis kit (Thermo Fisher Scientific, Waltham, MA, USA). For RT-PCR process, SYBR Green master mix (CloudSeq, Shanghai, China) was additionally employed. Primer 5.0 software (PREMIER Biosoft, Palo Alto, CA, USA) was used to design all primer sequences for circRNA targets as well as the housekeeping gene, i.e., glyceraldehyde-3-phosphate dehydrogenase (GAPDH). All primers were purchased from CloudSeq Biotechnology (Shanghai, China). The primer sequences are shown in Table 1. All raw data were acquired by QuantStudio 5 Real-Time PCR System (Thermo Fisher Scientific, Waltham, MA, USA), and further 2^–ΔΔ*Ct*^ method was applied to show the relative expression of these circulating circRNAs. Given that most of differentially expressed circRNA are novel, we used their chromosome locations to name them.

**TABLE 1:** The primer sequences for circular RNA (circRNA) identification.

	Genes		Sequences
1	chr11:74154652–74159524–	Forward	CATCTCCTACGCTTGCCTGA
		Reverse	CCAGAGAAACAAAGTGGCACG
2	chr12:45412398–45412635–	Forward	GAGACGAACCCAACCTGGTG
		Reverse	CGTGCCCTCCAAAATTGTACC
3	chr4:208359914–208362182+	Forward	ACTGGTGTGAATACTCGGCG
		Reverse	CTGTATGGGGCAATTCCGGT
4	chr7:137630261–137648924–	Forward	TCATGGGCAGTGGGATCTTG
		Reverse	GCAGTGAATAGAGATGCCCGA
5	chr15:20088296–20092102+	Forward	TGTGTGAGGCCTTGGTTTGA
		Reverse	CCACAAGTCCGTATCTTTGGC
6	chr1:175098934–175134845–	Forward	ACCTGGGCAAGGAATTCACC
		Reverse	GTGGTACTTGTGAGCCAGGG
7	chr1:175099657–175128203–	Forward	TCCACTGTGACAAGCTGCAT
		Reverse	GAATTCCTTGCCCAGGTGGT
8	chr1:175104454–175134845–	Forward	TTATGATGGGCCACCACCTG
		Reverse	GTGGTACTTGTGAGCCAGGG
9	chr6:6280974–6281188+	Forward	ACTCCTGGAGAACTCGGGAT
		Reverse	CCAGCTGCTACTTGCTCAGT
10	chr4:225251864–225254087+	Forward	GGAGCTGGAGAAGGACTTGG
		Reverse	CCTCTTGCCATTGTCCGTGA
11	GAPDH	Forward	GACATGCCGCCTGGAGAAAC
		Reverse	AGCCCAGGATGCCCTTTAGT

### Construction of circRNA-miRNA-mRNA network

Considering the importance of functional interactions among circRNAs, mRNAs and miRNAs during the gene regulation, the upstream as well as the downstream on the genome of these differential expressed circRNAs were extended in order to find functional genes near these circRNAs. To this end, corresponding miRNAs were predicted through employing miRNA target prediction software miRanda^6^ based on these differentially expressed circRNAs, and then potential mRNAs were further anticipated by applying Targetscan^7^ as well as miRDB^8^ (Sun et al., 2020). In the end, circRNA-miRNA-mRNA interaction network was established and visualized *via* Cytoscape^9^ (Zhao et al., 2021), where these differentially expressed circRNAs from circulating exosomes were centered.

### Statistical analysis

GraphPad Prism 8 software was utilized to make statistical analyses for these experimental results. Data were shown as means ± standard deviation (SD) from more than three statistical independent experiments. To compare the statistical significances among different groups, *t*-test of two independent samples was utilized, respectively. When *P*-value < 0.05, the difference was regarded as statistically significant. Especially, *P < 0.05, ^**^P < 0.01, ^***^P < 0.001, ^****^P < 0.0001.

## Results

### Expression profile analysis of circular RNAs derived from circulating exosomes

**FIGURE 1:**
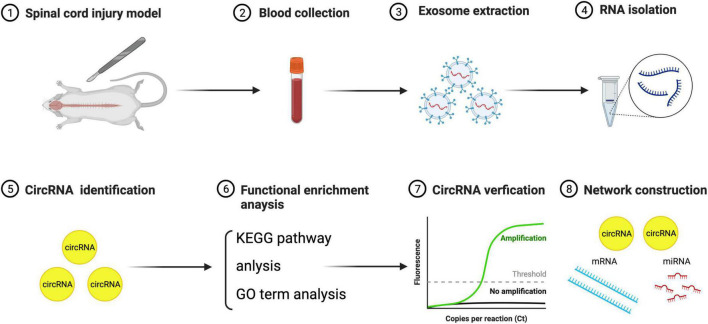
The flowchart of this study.

The experimental design of this study was shown in Figure 1. Part data of preliminary work including SCI rat model establishment and exosome identification were presented in our recently published paper (Li et al., 2023).

**FIGURE 2:**
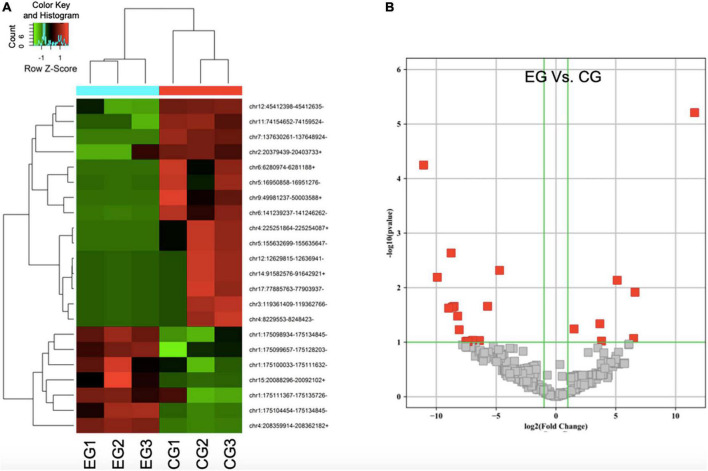
The expression profile of differentially expressed circular RNAs (circRNAs) in spinal cord injury (SCI) rats is identified. **(A)** Heat map of differentially expressed circRNAs from circulating exosomes. **(B)** Two-dimensional presentation for cluster analysis of differentially expressed circRNAs from circulating exosomes. Red represents relatively high expression, green represents relatively low expression, and black represents the average expression. *P* < 0.05, fold change > 2.0.

Through enrichment analyses, a total of 709 types of circRNAs were found to be downregulated whereas 346 kinds of circRNAs were shown to be upregulated in SCI rats in comparison with the control rats. In this experiment, three SCI rats and plus three control rats were included. Through the normalization as FPKM, the heat map of differentially expressed circRNAs from circulating exosomes including 15 kinds of downregulated circRNAs and 7 kinds of upregulated circRNAs was especially visualized (Figure 2A), suggesting that there exists a significantly different expression pattern of circulating exosomal circRNAs between SCI rats and the control rats (*P* < 0.05). In a similar vein, the volcano plots demonstrated consistent changes of differentially expressed circRNAs in SCI rats (Figure 2B). Taken together, these data give the first impression that some circulating circRNAs display differential expression in SCI rats.

### Enrichment analyses of potentially upregulated circular RNAs

**FIGURE 3:**
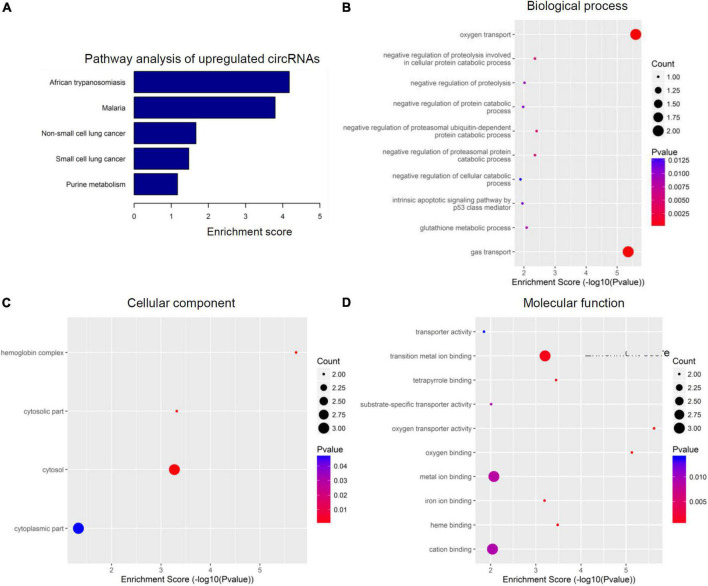
Kyoto encyclopedia of genes and genomes (KEGG) pathway analysis and gene ontology (GO) function analysis of upregulated circular RNAs (circRNAs) from circulating exosomes. **(A)** KEGG pathway analysis of upregulated circRNAs from circulating exosomes. **(B–D)** GO function analysis of upregulated circRNAs from circulating exosomes. **(B)** Biological process analysis of upregulated circRNAs from circulating exosomes. **(C)** Cellular component analysis of upregulated circRNAs from circulating exosomes. **(D)** Molecular function analysis of upregulated circRNAs from circulating exosomes.

**FIGURE 4:**
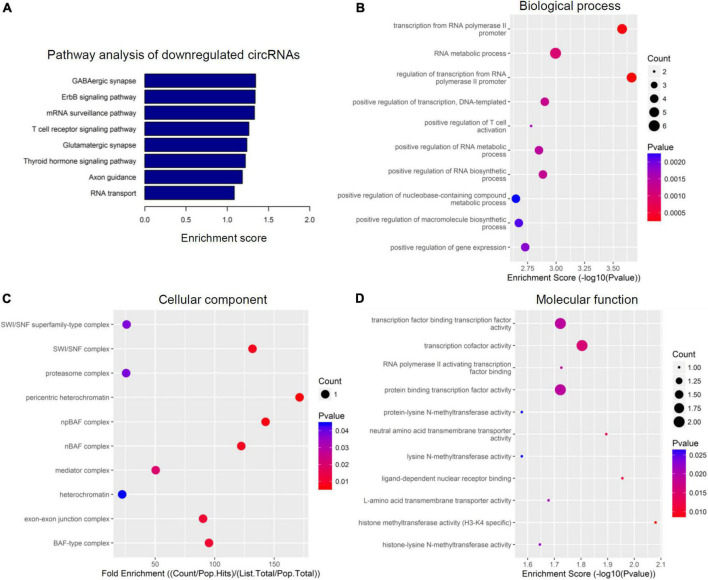
Kyoto encyclopedia of genes and genomes (KEGG) pathway analysis and gene ontology (GO) function analysis of downregulated circular RNAs (circRNAs) from circulating exosomes. **(A)** KEGG pathway analysis of downregulated circRNAs from circulating exosomes. **(B–D)** GO function analysis of downregulated circRNAs from circulating exosomes. **(B)** Biological process analysis of downregulated circRNAs from circulating exosomes. **(C)** Cellular component analysis of downregulated circRNAs from circulating exosomes. **(D)** Molecular function analysis of downregulated circRNAs from circulating exosomes.

To further speculate their specific functional activities and involved signaling pathways in the context of SCI, both GO analysis and KEGG analysis were next carried out in this study. Figures 3, 4 together demonstrated the top enriched GO terms and KEGG pathways for these differentially expressed cicRNAs. Especially, upregulated circRNAs were analyzed in Figure 3, and downregulated circRNAs were analyzed in Figure 4. These results together provide an overview of these probably involved cellular components, biological processes, and molecular functions as well as signaling pathways for differentially expressed circRNAs in SCI rats (Figures 3, 4), which may offer some meaningful indications for future research.

The top five enriched biochemical pathways for upregulated circRNAs were African trypanosomiasis, Malaria, non-small lung cancer, small cell lung cancer, and purine metabolism, as demonstrated by KEGG pathway analysis based on located genes (Figure 3A). On the other hand, GO enrichment analysis showed that these upregulated circRNAs were enriched in plenty of biological processes associated with inflammation, cellular components, and molecular functions. The top five enriched terms of biological process were oxygen transport, negative regulation of proteolysis involved in cellular protein catabolic process, negative regulation of proteolysis, negative regulation of protein catabolic process, and negative regulation of proteasomal ubiquitin-dependent protein catabolic process (Figure 3B). The top four enriched terms of cellular component were hemoglobin complex, cytosolic part, cytosol, and cytoplasmic part (Figure 3C). At last, oxygen transporter activity, oxygen binding, heme binding, tetrapyrrole binding, and transition metal ion binding were the top five enriched terms of molecular function (Figure 3D). Of special interest, the potential involvement of some circRNAs in tumors may indicate the potency of circRNA-elicited microenvironment changes in inflammatory processes.

### Enrichment analyses of potentially downregulated circular RNAs

The top eight enriched biochemical pathways were GABAergic synapse, ErbB signaling pathway, mRNA surveillance pathway, T cell receptor signaling pathway, glutamatergic synapse, thyroid hormone signaling pathway, axon guidance and RNA transport, as demonstrated by KEGG pathway analysis based on located genes (Figure 4A). In addition, GO enrichment analysis showed that these downregulated circRNAs were enriched in plenty of biological processes associated with inflammation, cellular components, and molecular functions. The top five enriched terms of biological process were transcription from RNA polymerase II promoter, RNA metabolic process, regulation of transcription, DNA-templated, and positive regulation of T cell activation (Figure 4B). The top five enriched terms of cellular component were SWI/SNF superfamily-type complex, SWI/SNF complex, proteasome complex, pericentric heterochromatin, and npBAF complex (Figure 4C). At last, transcription factor binding transcription factor activity, transcription factor activity, RNA polymerase II activating transcription factor binding, protein binding transcription activity, and protein-lysine *N*-methyltransferase activity were the top five enriched terms of molecular function (Figure 4D). In combination with data for upregulated circRNAs, these bioinformatics data together suggest there are different pathways and functions involved for these dysregulated circRNAs.

### Verification of differentially expressed circular RNAs through reverse transcription-polymerase chain reaction

**FIGURE 5:**
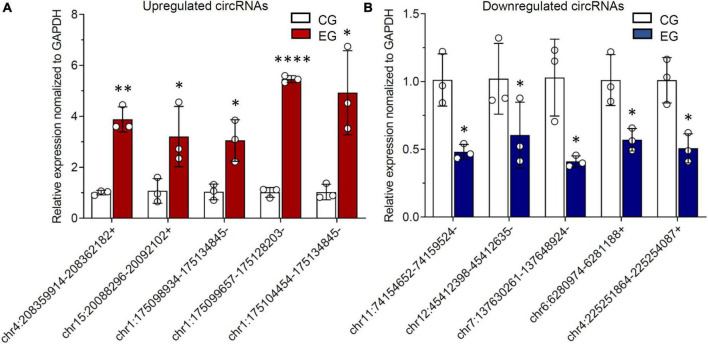
Differentially expressed circular RNAs (circRNAs) from circulating exosomes are verified by reverse transcription-polymerase chain reaction (RT-PCR). **(A)** The relative expression levels of upregulated circRNAs, including chr4:208359914–208362182+, chr15:20088296–20092102+, chr1:175098934– 175134845–, chr1:175099657– 175128203–, and chr1:175104454–175134845–. **(B)** The relative expression levels of downregulated circRNAs, including chr11:74154652– 74159524–, chr12:45412398– 45412635–, chr7:137630261– 137648924–, chr6:6280974–6281188+, and chr4:225251864–225254087+. * *P* < 0.05, ^**^ *P* < 0.01, ^****^ *P* < 0.0001.

As addressed in the above part, several significantly upregulated and downregulated circRNAs were preliminarily selected through RNA sequencing (Figure 2). Later on, the potential roles of these circulating circRNAs in SCI rats were predicted *via* KEGG and GO analyses (Figures 3, 4). Therefore, we would like to take advantage of qRT-PCR assay, to verify whether these candidate circRNAs are significantly dysregulated indeed. It turned out that there were a total of ten kinds of differentially expressed circRNAs, which were further verified by qRT-PCR in this study. Quantification results displayed that the relative expression of chr4:208359914–208362182+, chr15:20088296–20092102+, chr1:175098934– 175134845–, chr1:175099657– 175128203–, and chr1:175104454– 175134845–, was significantly elevated in SCI rats in comparison with the control rats (Figure 5A). By contrast, the relative levels of chr11:74154652– 74159524–, chr12:45412398– 45412635–, chr7:137630261– 137648924–, chr6:6280974–6281188+, and chr4:225251864–225254087+ were dramatically diminished in SCI rats compared to the controls (Figure 5B). Moreover, the detailed information of these ten candidate circRNAs is summarized in Table 2. Given that most of differentially expressed circRNA are novel and the gene symbol of three kinds of circRNA is the same, we named them according to their chromosome locations. Relevant predicted pathway information of each circRNA can be checked through circBase^10^ (Table 2). These quantitative results not only provide some promising targets for future *in vivo* and *in vitro* research, but also validate the expression profile of circulating circRNAs indicated by RNA sequencing.

**TABLE 2:** Basic information of differentially expressed circular RNAs (circRNAs).

Source	Position	Genomic length	Strand	Best transcript	Gene symbol	Catalog	Regulation
PMID: 25714049	chr11:74154652–74159524	4,872	–	XM_003 751063	Lrch3	Exonic	Down
PMID: 25714049	chr12:45412398–45412635	237	–	XM_003 751198	Med13l	Exonic	Down
PMID: 25714049	chr4:208359914–208362182	2,268	+	NM_0011 06614	Setd5	Exonic	Up
PMID: 25714049	chr7:137630261–137648924	18,663	–	NM_13 8832	Slc38al	Exonic	Down
Novel	chr15:20088296–20092102	3,806	+	NM_02 1774	Fhit	Intronic	Up
Novel	chr1:175098934–175134845	35,911	–	NM_03 3234	Hbb	Sense overlapping	Up
Novel	chr1:175099657–175128203	28,546	–	NM_03 3234	Hbb	Sense overlapping	Up
Novel	chr1:175104454–175134845	30,391	–	NM_03 3234	Hbb	Sense overlapping	Up
Novel	chr6:6280974–6281188	214	+	XM_592846	Loc103 692	Exonic	Down
Novel	chr4:225251864–225254087	2,223	+	–	–	Intergenic	Down

### Construction of circRNA-miRNA-mRNA interaction network to predict functions of differentially expressed circular RNAs

**FIGURE 6:**
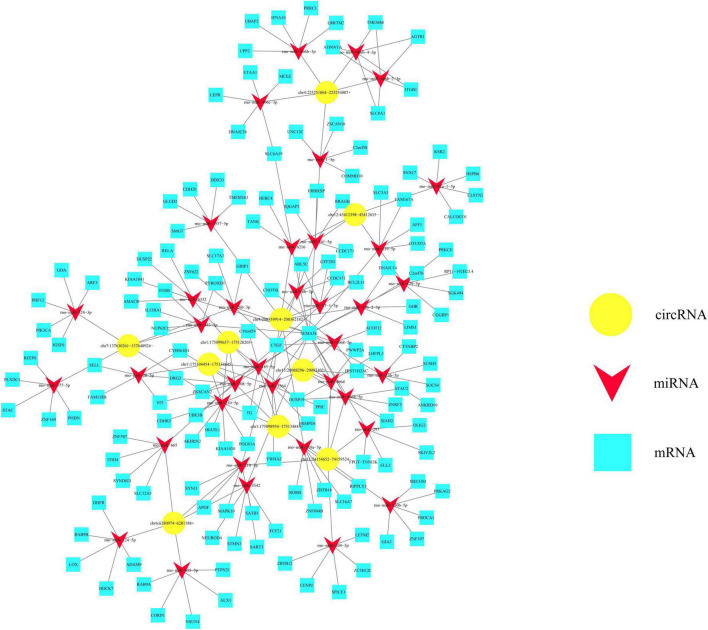
Potential value of differentially expressed circular RNAs (circRNAs) from circulating exosomes is predicted based on the circRNA-miRNA-mRNA interaction network. This interaction network is centered by 10 kinds of differentially expressed circRNAs, as indicated by yellow polygons. These circRNAs could co-express and interact with 37 miRNAs which are indicated by red triangles, and 145 mRNAs which are indicated by blue boxes.

Based on ceRNA mechanism, we would like to take advantage of circRNA-miRNA-mRNA interaction network to anticipate potential functions of circRNAs in the injured spinal cord. As shown in Figure 6, five kinds of upregulated circRNAs as well as five kinds of downregulated circRNAs verified by RT-PCR were chosen and centered in this network. These circRNAs could co-express and interact with 37 miRNAs which are indicated by red triangles and 145 mRNAs which are indicated by blue boxes (Figure 6). All these upregulated circRNAs had four to six target miRNAs, as exemplified by chr4:208359914–208362182+ which has five target miRNAs including rno-miR-26b-3p, rno-miR-17-1-3p, rno-miR-3068-5p, rno-miR-3564, and rno-miR-20b-3p (Figure 6). In the meanwhile, these downregulated circRNAs such as chr11:74154652– 74159524–, chr12:45412398– 45412635–, chr7:137630261– 137648924–, chr6:6280974–6281188+, and chr4:225251864–225254087+ had three to six target miRNAs generally (Figure 6). Thus, these bioinformatics data together imply the potential important functions of the above-mentioned circulating circRNAs in regulating other genes.

## Discussion

In the current study, bioinformatics analyses of rat specimens revealed 709 downregulated circRNAs and 346 upregulated circRNAs from circulating exosomes in SCI rats in comparison with the control rats, and RT-RCR further confirmed their differential expression. These results together highlight the potential involvement of exosomal circRNAs in injured spinal cord.

In fact, there have been plenty of published studies revealing differentially expressed circRNAs during different stages of SCI (Qin et al., 2019; Wu et al., 2019; Liu et al., 2020; Peng et al., 2020). Liu et al. (2020) found that 1101 circRNAs were upregulated and 897 circRNAs were downregulated at the immediate stage of SCI. Furthermore, they identified eight out of ten candidate circRNAs through RT-PCR, such as rno_circRNA_011494 and rno_circRNA_009608, which displayed different levels of dysregulated expression in SCI rats in comparison with the control group, suggesting several possible targets for early intervention of SCI (Liu et al., 2020). In a similar vein, Wu et al. (2019) displayed a more broad expression profile of spinal cord-derived circRNAs at seven time points including immediate and chronic phases. Of note, they especially focused on exonic circRNA_01477, and demonstrated silencing of circRNA_01477 ameliorated astrocyte proliferation and migration through *in vitro* functional assay. Mechanistically, circRNA_01477 depletion was associated with significantly reduced miRNA-423-5p expression (Wu et al., 2019), indicating that circRNA_01477/miR-423-5p may contribute to SCI progression. Taken together, the important role of circRNAs in SCI has been well established.

In the functional sense, circRNAs have been extensively implicated in SCI through multiple mechanisms, as systematically reviewed by Sámano et al. (2021) recently. Through SCI mouse as well as rat models, circular RNAs have been found to regulate proliferation and migration of vascular endothelial cells, angiogenesis, neuronal cell apoptosis, and neuroinflammation, based on ceRNA pattern (Zhao et al., 2020; Chen J. et al., 2021; Xie et al., 2021; Ye et al., 2021). Among these reported circRNAs, circRNA_014301 showed a pro-inflammatory effect and a pro-apoptotic property upon lipopolysaccharide (LPS) stimulation, which could be reversed by its silencing in PC12 cells (Xie et al., 2021). It suggests the suppression of circRNA_014301 may be a promising strategy to control SCI progression. By contrast, circ-HIPK3 was found to be downregulated in SCI rats and further circ-HIPK3/miR-558/DPYSL5 axis protects against neuronal cell apoptosis in injured spinal cord (Zhao et al., 2020). Moreover, Chen Y. et al. (2021) found another novel circRNA-2960, which could aggravate inflammatory response and induce apoptosis through downregulating miRNA-124 at the lesion site. More recently, Qi et al. (2022) identified circ-Ctnnb1 as a potent regulator of neuronal injury in SCI through the Wnt/β-catenin signaling pathway. Collectively, there have been plenty of well-studied circRNAs in the context of SCI so far, and these circRNAs functionally contribute to SCI progression and even recovery.

However, the majority of current mechanistic studies utilized the spinal cord as the major specimen source and thereby investigated spinal cord-derived circRNAs after injury (Liu et al., 2020; Peng et al., 2020; Chen J. et al., 2021), as discussed in the above part. In addition to the spinal cord itself, circulating mediators from blood including inflammatory factors and bioactive lipids are also considered as important regulators for SCI development (Francos-Quijorna et al., 2017; David and López-Vales, 2021). On the one hand, sudden injury of spinal cord could induce inflammation, a complex response that contributes to secondary damage and even severe functional loss (Hellenbrand et al., 2021; Liu et al., 2021). On the other hand, exosomal circRNAs can participate in multiple inflammatory processes, such as NLRP3 inflammasome activation (Li M. et al., 2020), macrophage polarization and inflammation (Song et al., 2022), autoimmune response (Lodde et al., 2020). Given the importance of circRNAs in inflammation, we are curious about whether circulating circRNAs contribute to SCI. However, there are no relevant data yet. Therefore, our study for the first time addressed the potential value of circulating exosomal circRNAs in SCI progression. Newly identified differentially expressed circRNAs would be promising candidates for early intervention and treatment of SCI.

Of note, we applied enrichment analyses including KEGG pathway, GO, and interaction network prediction in this study, which are well established to anticipate potent genes involved in SCI (Li Z. et al., 2020; Chen Q. et al., 2021). Top enriched biochemical pathways and GO terms of dysregulated circRNAs could provide us some meaningful hints for further mechanistic study. More importantly, the circRNA-miRNA-mRNA interactive network sheds light on the functional involvement of circRNAs in SCI development (Peng et al., 2020; Tong et al., 2021; Wang et al., 2021). Our data demonstrated these differentially expressed circRNAs could co-express and interact with 37 miRNAs and 145 mRNAs. Previous studies have identified several circRNA-miRNA pairs in SCI, such as circAbca1/miR-135b, circRNA_01477/miR-423-5p, circ-HIPK3/miR-558 (Wu et al., 2019; Zhao et al., 2020; Wang et al., 2021). In this study, we also constructed the interaction network, which was centered by 10 kinds of differentially expressed circRNAs. These circRNAs could co-express and interact with 37 types of miRNAs, and 145 types of mRNAs. In combination with the previously published study, these bioinformatics results together kick off functional research of circulating exosomal circRNAs in this field.

In the meanwhile, we acknowledge that there exist several limitations in our study. Even though the expression profile of circulating circRNAs has been mapped in this study, we did not go deeper considering any kind of circRNA. Apparently, *in vitro* functional assays and pharmacological investigations are called for further mechanistic exploration. In addition, concerning differences in circRNAs among species, the clinical significance of human circRNAs in patients should be clarified. Furthermore, solid experimental evidence for the interaction between circRNAs and corresponding miRNAs would bring new avenues for targeting SCI pathological process.

In conclusion, our data identify several circulating exosomal circRNAs in SCI rats, which show a differential expression pattern. These circRNAs are predicted to be involved in different pathways and functional processes, and thereby contribute to SCI development, providing some potential targets for future research on SCI.

## Data availability statement

The datasets presented in this study can be found in online repositories. The names of the repository/repositories and accession number(s) can be found below: https://www.ncbi.nlm.nih.gov/geo; GSE213561.

## Ethics statement

This animal study was reviewed and approved by the Animal Care and Use Committee of Tijian Hospital.

## Author contributions

JL, CZ, and ZW designed and conceptualized this study. JL and CZ wrote the manuscript. FL and ZW further polished the manuscript. All authors prepared and performed experiments, collected, analyzed, and interpreted data, read, and approved the final manuscript.

## Footnotes

1
https://cutadapt.readthedocs.io/

2
http://daehwankimlab.github.io/hisat2/

3
http://www.ensembl.org/

4
http://www.genome.jp/kegg

5
http://www.geneontology.org

6
http://cbio.mskcc.org/microrna_data/miRanda-aug2010.tar.gz

7
https://www.targetscan.org/vert_80/

8
http://www.mirdb.org/

9
https://cytoscape.org/

10
http://www.circbase.org/
